# Going back to “basics”: Harlow’s learning set task with wolves and dogs

**DOI:** 10.3758/s13420-024-00631-6

**Published:** 2024-05-23

**Authors:** Dániel Rivas-Blanco, Tiago Monteiro, Zsófia Virányi, Friederike Range

**Affiliations:** 1 Domestication Lab, Department of Interdisciplinary Life Sciences, Konrad Lorenz Institute of Ethology, University of Veterinary Medicine Vienna, Vienna, Austria; 2 William James Center for Research, University of Aveiro, Aveiro, Portugal; 3 Comparative Cognition, Messerli Research Institute, University of Veterinary Medicine Vienna, Vienna, Austria; 4 Medical University Vienna, University of Vienna, Vienna, Austria

**Keywords:** Associative learning, Dogs, Domestication, Learning set, Serial learning, Reversal learning, Wolves

## Abstract

To survive and reproduce, animals need to behave adaptively by adjusting their behavior to their environment, with learning facilitating some of these processes. Dogs have become a go-to model species in comparative cognition studies, making our understanding of their learning skills paramount at multiple levels, not only with regards to basic research on their cognitive skills and the effects of domestication, but also with applied purposes such as training. In order to tackle these issues, we tested similarly raised wolves and dogs in a serial learning task inspired by Harlow’s “learning set.” In *Phase 1*, different pairs of objects were presented to the animals, one of which was baited while the other was not. Both species’ performance gradually improved with each new set of objects, showing that they “learnt to learn,” but no differences were found between the species in their learning speed. In *Phase 2*, once subjects had learned the association between one of the objects and the food reward, the contingencies were reversed and the previously unrewarded object of the same pair was now rewarded. Dogs’ performance in this task seemed to be better than wolves’, albeit only when considering just the first session of each reversal, suggesting that the dogs might be more flexible than wolves. Further research (possibly with the aid of refined methods such as computer-based tasks) would help ascertain whether these differences between wolves and dogs are persistent across different learning tasks.

**Supplementary Information:**

The online version contains supplementary material available at 10.3758/s13420-024-00631-6.

## Introduction

Learning is the process by which an animal acquires new knowledge, behaviors, or skills (Gross, 2012), and is shown to be crucial in the way animals interact with their environment: with past experiences informing current behavior, as well as information from novel opportunities (e.g., a new resource on which to feed) or risks (e.g., a poisonous animal that should be avoided) being integrated in their decision-making processes and assisting survival.

One of the most common forms of learning is instrumental learning (also known as operant conditioning), defined as the association between behavior and the reinforcement or punishment that results from it (Mazur, 2016), often through a trial-and-error process (e.g., making an association between opening a garbage bin and finding food inside; Bitterman, 1969). Instrumental learning is a highly conserved skill throughout taxa, being present in some capacity in animals ranging from nematodes to vertebrates (Gourgou et al., 2021; Pavlov, 1960). As such, it is particularly useful to design experimental paradigms that can easily be adapted across species with different cognitive abilities and *Umwelten* in order to compare their abilities (Uexküll & Mackinnon, 1926).

Serial learning experiments (i.e., experiments in which different learning events or opportunities succeed after each other) open the possibility to study more complex behaviors, such as discriminations of quantity and ordinal position (Terrace, 2005). Perhaps most importantly, however, is the study of serial expertise (as defined by Terrace, 2005): an animal’s ability to gradually increase the speed at which they acquire associations in a given task (or, in simpler terms, their capability of “learning to learn”; Shettleworth, 2010). This paradigm, in turn, provides the conceptual basis for reversal learning tasks, in which the task contingencies are reversed every few trials (i.e., the previously negative stimulus would become the positive one and vice versa; for examples see Bond et al. (2007), Chidambaram et al. (2024), Chittka (1998), Eimas (1966), Mackintosh (1965), and Williams (1967)). These latter experiments are meant to measure an animal’s ability to extinguish previously learned associations and acquire new ones, something that has been used as a measure of behavioral flexibility (Audet & Lefebvre, 2017; Izquierdo et al., 2017).

A classic paradigm used to test serial expertise is the “learning set” paradigm – described by Harlow in 1949. In that study, rhesus macaques were initially tested in a serial discrimination task in which 344 pairs of different objects were presented. Of these, one object of each pair was considered the "positive" stimulus (which would grant a food reward upon being chosen by the subject), and the other, the "negative" one (which would beget no reward). Each object pair was presented several times so that the subject could learn the association between choosing the positive stimulus and getting the reward (Harlow, 1949). The subjects showed a gradual improvement throughout object pairs, getting an almost 100% success rate at trial 2 by the end of the experiment (i.e., after experiencing hundreds of object pairs). This showed that macaques were not only able to associate choosing the positive stimulus with getting the food reward, but to transfer the knowledge of the contingencies of the test to new iterations of it, hence "learning to learn" (Shettleworth, 2010). After being tested in this initial task, the same subjects participated in a subsequent reversal learning experiment. As was the case with the preceding experiment, an almost 100% success rate at trial 2 was reached for the last reversals. Interestingly, even though this second experiment was, in principle, a more complex task, the subjects showed consistently better performance than in the previous experiment, possibly as a result of generalizing from the serial discrimination task to the reversal (i.e., animals not only “learned to learn” within the same task, but also across tasks).

Similar experiments were later performed on other species (e.g., chimpanzees (Hayes et al., 1953), marmosets (Miles & Meyer, 1956), rats (Koronakos & Arnold, 1957), cats (Warren, 1966), pigeons (Zeigler, 1961), and blue jays and crows (Hunter, 1970). However, the subjects rarely achieved that nigh-perfect level of success the rhesus macaques reached at trial 2 of each new object pair. In many cases, this could be attributed to the reduced number of object pairs the animals were trained to discriminate. Koronako’s and Arnold’s study (1957) shows that only a small fraction of subjects (five of the 20 rats tested) were able to achieve 80% of correct choices for all eight sets presented, although the compounding effect of each successive discrimination was not taken into consideration (i.e., whether they learnt to learn was not tested for, as no analyses were performed on their improvement with each successive set). Similarly, pigeons (Zeigler, 1961) reached around 60% of successful choices at second trial and around 70% on the last trial of the last few sets (80% when taking only the data from the most successful subjects), and although pigeons were exposed to many more sets of items than the rats, set numbers were still considerably less than in Harlow’s original study (with 120 sets presented vs. 344 used in Harlow’s original experiment). After this original outburst of learning set experiments, later experiments focused on the “reversal” learning part of the paradigm, more as a measure of flexibility rather than of learning – often without being previously presented with a preceding serial discrimination task, different to the original experiment (Bond et al., 2007; Erdsack et al., 2022; Rayburn-Reeves et al., 2013).

One species with which this paradigm has been seldom used is dogs. This comes as a surprise, since knowing more about dogs’ instrumental learning skills would be crucial for at least two reasons: (1) to develop better-suited training practices, and (2) to support comparative cognition research. With regard to the former, working dog training (detection, guarding, support, etc.) hinges on our understanding of their instrumental learning skills (Concha et al., 2014; Deldalle & Gaunet, 2014; Helton, 2009). As for the latter, dogs have become one of the most popular study species in the field of animal cognition (particularly in the study of social cognition – e.g., Fugazza et al., 2016; Horowitz, 2009; Nagasawa et al., 2011; for reviews, see Bensky et al., 2013; Miklosi, 2007; Range & Marshall-Pescini, 2022b), so the entire field could greatly benefit from a thorough understanding of to what extent and in what way dogs’ performance in such experiments can be explained by instrumental learning processes (Dickinson, 2012).

Compared to studies on dogs’ social cognition, few of these have addressed how domestication may have affected their learning skills. A study by Frank and Frank (1987) in which both a serial discrimination and a reversal task were carried out showed that dogs outperformed wolves when it came to reversal learning (but not to the basic serial discrimination learning). However, it has been pointed out that these wolves’ performance may have been an artifact of them being uncomfortable with the testing setting, as they were socialized to humans to a limited degree. Indeed, later on this study was replicated with hand-reared wolves that outperformed not only their mother-reared counterparts, but also dogs, both in the reversal task but also on the serial discrimination (Frank, 2011). A reversal learning study was also carried out in dogs and wolves by Brucks et al. (2019), but they did not find any differences between the species. It remains unclear, then, whether there truly are differences between some of the learning skills of dogs and wolves.

One of the most relevant hypotheses has been put forward by Frank (2011), suggesting that domestication largely increased the tractability of dogs by endowing them with a sensitivity to a broader band of stimuli (in particular, to arbitrary cues with no functional connection with the outcome) and with sufficient behavioral plasticity, preparing them to fulfill different jobs (e.g., police and sheep herding dogs). If so, one would expect dogs to be faster in Harlow’s serial learning tasks, in comparison with wolves (their closest-living relatives – Ostrander et al., 2019), which did not undergo the domestication process. Importantly, this is likely to include better performance in both phases of the task: higher responsiveness to arbitrary stimuli likely facilitates object-food associations whereas higher behavioral plasticity likely enables more flexible reversals.

Different to Frank’s tractability hypothesis, other findings have shown that wolves are, in general, more motivated and persistent than dogs when it comes to object manipulation and working for food (Marshall-Pescini et al., 2017; Rao et al., 2018), which might give them an advantage in problem-solving tasks (Chow et al., 2016). Accordingly, it would be expected that wolves would outperform dogs in experiments testing their learning abilities by virtue of them being more engaged in the task (persistence hypothesis). This higher persistence, however, could prove detrimental in reversal learning tasks, as it could hamper their ability to switch strategies when the reward contingencies are swapped.

Furthermore, as discussed by Sih et al. (2011), human-created environments are particularly volatile, and should select for higher levels of behavioral flexibility (we will refer to this hypothesis as the “human-driven flexibility hypothesis”). Over the process of domestication, dogs associated first with hunter gatherers, then with humans living in small settlements, and finally with humans living in larger villages and cities. These ancient dogs (and, similarly, modern-day free-ranging dogs) may have access to plentiful food sources, that may nonetheless vary considerably across time and space (e.g., human refuse, garbage bins, fecal matter, etc.; Atickem et al., 2009; Hughes & Macdonald, 2013; Lord et al., 2013; Sarkar et al., 2023; Vanak & Gompper, 2009). In contrast, wolves are cooperative hunters living in relatively predictable (but also relatively scarce) environments (Mech et al., 2015; Mech & Boitani, 2007). As such, it would follow that dogs’ reliance on the changing human-shaped environment should make dogs more capable of discarding associations that are no longer adaptive, making them more flexible, even if not necessarily faster, learners than wolves.

This study aimed to explore and compare dogs’ and wolves’ performance on a task inspired by Harlow’s (1949) “learning set” task. Similar to Harlow’s study, we used several object pairs for the first serial discrimination experimental phase (i.e., the one in which one object was associated with a food reward) and, after a learning criterion was met, the subjects faced another experimental phase in which the same pair of items switched back and forth between baiting one item or the other, respectively. Critically, dogs and wolves that participated in this study were raised and kept under similar circumstances, to ease cross-species comparisons. Through this study, we endeavor to shed light on the effects of domestication on dogs’ and wolves’ learning capabilities.

## Methods

### Subjects

*Phase 1* started with 17 wolves (*Canis lupus*; 15.2 ± 2.4 months at first session) and 22 dogs (*Canis familiaris;* 14.2 ± 2.4 months at first session). A subset of these animals was also tested in *Phase 2* (eight wolves (28.4 ± 3.5 months at the first session – see Online Supplementary Material (OSM) Table1) and seven dogs (36 ± 4.6 months at the first session of acquisition).

**Table 1:** Statistical models

1: Phase 1, session of success	
1.1: Full model	Outcome (session) ~ Species * Session# + Species * Set# + (1 + Session# + Set# ‖ Subject) + (1 + Species ‖ Set# / SessionID)
1.2: Reduced model	Outcome (session) ~ Species + Session# + Set# + (1 + Session# + Set# ‖ Subject) + (1 + Species ‖ Set# / SessionID)
1.3: Null model	Outcome (session) ~ Session# + Set# + (1 + Session# + Set# ‖ Subject) + (1 + Species ‖ Set# / SessionID)
2: Phase 1, trial of success at first session	
2.1: Full model	Outcome (trial; 1st session of set) ~ Species * Trial# + Species * Set# + (1 + Trial# + Set# ‖ Subject) + (1 + Species ‖ Set# / TrialID)
2.2: Reduced model	Outcome (trial; 1st session of set) ~ Species + Trial# + Set# + (1 + Trial# + Set# ‖ Subject) + (1 + Species ‖ Set# / TrialID)
2.3: Null model	Outcome (trial; 1st session of set) ~ Trial# + Set# + (1 + Trial# + Set# ‖ Subject) + (1 + Species ‖ Set# / TrialID)
3: Phase 2, session success	
3.1: Full model	Outcome (session) ~ Species * Session# + Species * Reversal# + (1 + Session# + Reversal# ‖ Subject) + (1 + Species ‖ Reversal# / SessionID)
3.2: Reduced model	Outcome (session) ~ Species + Session# + Reversal# + (1 + Session# + Reversal# ‖ Subject) + (1 + Species ‖ Reversal# / SessionID)
3.3: Null model	Outcome (session) ~ Session# + Reversal# + (1 + Session# + Reversal# ‖ Subject) + (1 + Species ‖ Reversal# / SessionID)
4: Phase 2, trial of success at first session	
4.1: Full model	Outcome (trial; 1st session of set) ~ Species * Trial# + Species * Reversal# + (1 + Trial# + Reversal# ‖ Subject) + (1 + Species ‖ Reversal# / TrialID)
4.2: Reduced model	Outcome (trial; 1st session of set) ~ Species + Trial# + Reversal# + (1 + Trial# + Reversal# ‖ Subject) + (1 + Species ‖ Reversal# / TrialID)
4.3: Null model	Outcome (trial; 1st session of set) ~ Trial# + Reversal# + (1 + Trial# + Reversal# ‖ Subject) + (1 + Species ‖ Reversal# / TrialID)

**Fig. 1:**
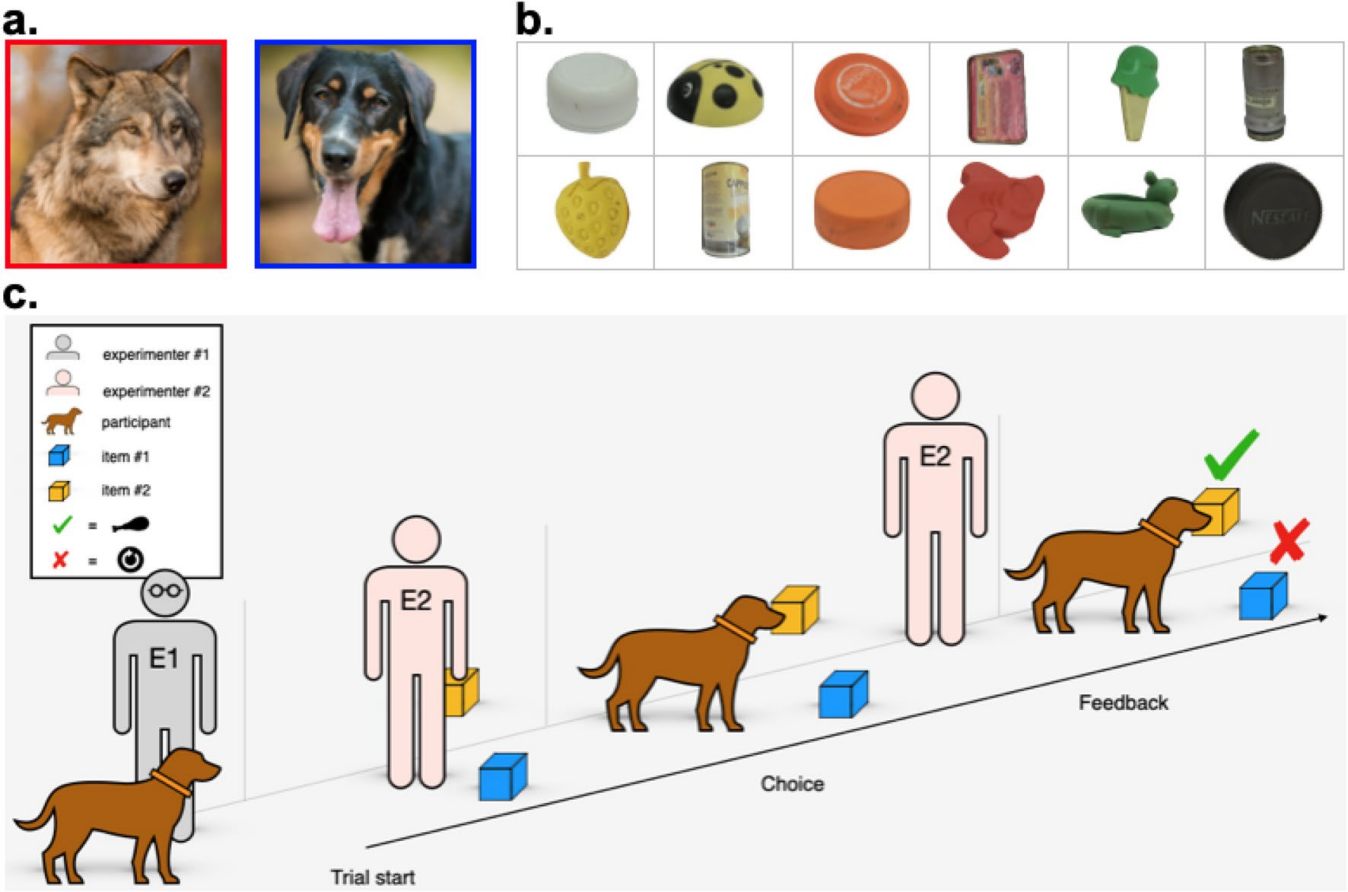
**(a)** Subjects: Wolves (*Canis lupus*) and dogs (*Canis familiaris*) raised and kept in similar environments participated in this study. See Online Supplementary Material (OSM) Table 1 for details. **(b)** Stimuli: Example items used in the experiment. See OSM Table 2 for details. **(c)** Procedure: Two human experimenters (E1 and E2) were needed to run *Phases 1* and *2*. While E1 controlled the participants, E2 was responsible for stimuli placement and associated contingencies. The trials of both experimental phases consisted of three main parts: trial start, choice, and feedback. See *General procedure* for details

Both wolves and dogs (Fig. 1a) were raised in a similar environment. They were separated from their mothers at 10 days of age and then hand-raised by humans for 5 months. The pups were then integrated in packs with other adult conspecifics and housed in large 2,000–8,000m^2^ outdoor enclosures. All animals were trained to perform basic commands, participated regularly in behavioral experiments with unrelated contingencies and/or stimulus objects (e.g., string pulling tasks, pointing studies, social learning) in the same testing facility, and had daily interactions with the experimenters. A complete list of subject-related information can be found in OSM Table 1.

### Materials

Several pairs of items of different sizes and colors were presented to the subjects, all of them with some sort of crevice or gap under which a piece of high-quality reward (meat or sausage) could be placed. A comprehensive list of the items used can be found in OSM Table 2; example pictures can be found in Fig. 1b.

### Testing facility

Testing was conducted in a large indoor testing area of 5.4 x 9 m at the Wolf Science Center, in Austria. To reduce the possibility of the animals developing side bias, the starting position of the animals was changed on every trial within a session, and the objects were placed at different locations (i.e., moving to different areas within the testing chamber).

### General procedure

Both *Phase 1* and *Phase 2* shared the same basic procedure (Fig. 1c). Two experimenters (E1 and E2 hereafter) conducted the study. E1 called the animal to the starting position, asked the subject to sit down next to them, rewarded them with a food reward, and then held the animal by the collar. E2 baited the positive stimulus with a food reward (baiting was done with their back turned so that animals could not see it), and thoroughly smeared the second stimulus with food to prevent the animals from finding the food reward by their sense of smell alone. Then E2 moved to stand in front of E1 with their back facing the subject, holding both objects in their hands. The distance between E2 and the subject at the start of each trial would depend on the species, one long step (~1 m) for dogs and two to three steps for wolves due to the differences in size and speed between the species (except for one of the wolves – Nanuk, for which the distance was reduced to 1–1.5 m due to a sight impairment).

Once E2 was in position, E1 either stared down or closed her eyes to avoid cuing the animal inadvertently. E2 then stepped about 2–3 m forward and placed the two objects on the floor one after the other, around 1.5–2 m away from each other, by extending their arms and crouching, but keeping their body approximately at the same distance from both objects. After this, E2 took another step forward, leaving the objects behind them, with their back still facing the animal.

E1 then released the animal and the trial commenced. A subject would be considered to have made a choice when they came into contact with one of the objects. If the subject made the correct choice, they were allowed to eat the reward. However, if the subject made the incorrect choice, E1 would walk towards the reward under the positive stimulus and take it away before the animal could reach it (due to safety concerns, in cases in which E1 would not be able to take away the reward, E2 would do it instead). If the subject failed to make a choice, the trial was repeated until they did so. However, some subjects lost motivation during some sessions and failed to make a choice, regardless of the repetitions of that trial. In such cases, the session was aborted on that trial.

Each session consisted of ten trials; in five of them the positive item was on the right side, in five of them on the left side; no more than twice in a row on the same side. The order in which the items were placed on the floor was counterbalanced according to a predetermined assignment, with the positive stimulus presented first on some trials and second on others; this ordering was also not repeated more than twice in a row within the same session. Furthermore, the same combination of side and order of presentation was never repeated (i.e., if the positive stimulus was presented first on the right side in a given trial, the next trial in which the positive stimulus was placed on the right side, it would have to be presented second).

Sessions were carried out once a week, but larger gaps took place whenever other experiments were carried out (as well as any other disruptive events, such as health checks and the breeding season).

#### Phase 1: Serial discrimination learning

**Fig. 2:**
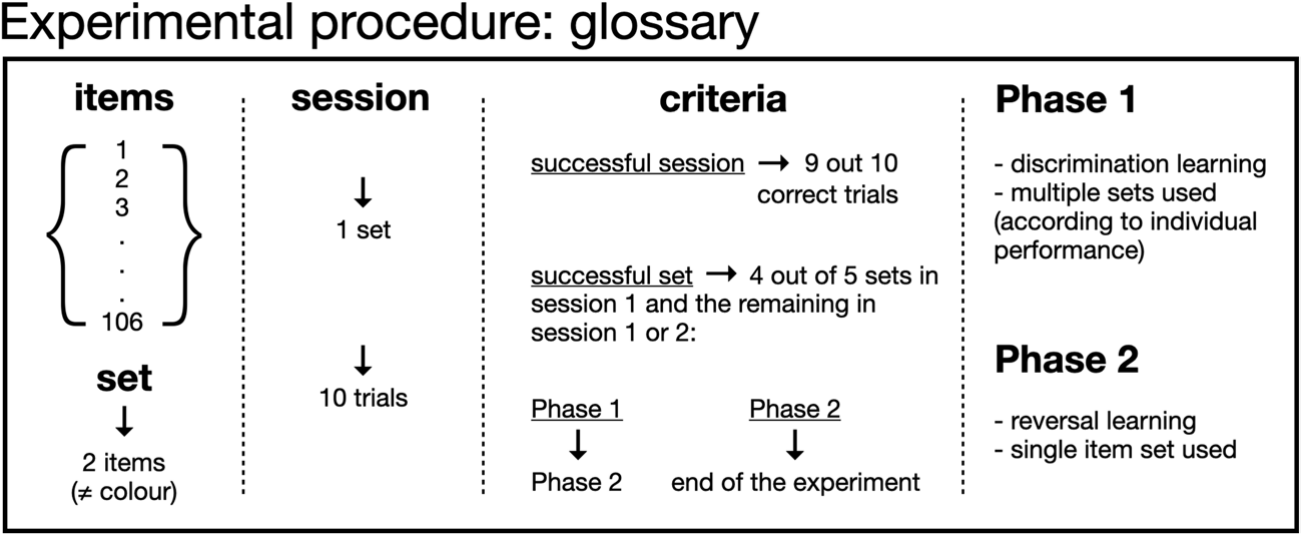
Glossary of experimental terms and key procedural milestones. There was a pool of 106 potential *items* (see Online Supplementary Material Table 1 for more details). A *set* was composed of two items of different color (see *Materials* for details). Each *session* included a single set of items and ten trials (see *General procedure* for details). In order to advance, animals needed to reach within- and across- session *success criteria*: nine out of ten correct trials moved animals within each experimental phase, and four out of five successful sets within the first session (and the remaining one successful within the first or second session) moved the animals to *Phase 2* (if they were in *Phase 1*) or the end of the experiment (if they were in *Phase 2*), respectively. In *Phase 1*, animals were trained to discriminate across different item sets, whereas in *Phase 2*, instead of introducing a new set, within-set contingencies would be swapped if a session was successful

During this phase, several sets of items were presented to the subjects, randomly matching different kinds of items without the same item being presented as part of more than one set for any given subject (see Fig. 2 and OSM Table 2 for the complete list of items used). Within sets, items were of different colors. Furthermore, color pairings used in a given set of items could not be repeated within the subsequent five sets. Whenever a subject was exposed to an item with a color that matched another item from a previous set, this item was of the opposite stimulus type (i.e., if a purple item was used as a positive stimulus in one of the sets, the next time a purple item was used for the same subject, it was the negative stimulus instead). This counterbalancing was done to prevent spontaneous biases from potentially arising within the subjects, as dogs have been shown to be able to discriminate between a variety of colors despite only having two different types of cone photoreceptor cells (Kasparson et al., 2013). Nonetheless, some of the items used were considered as “mixed” color (i.e., they did not have a unique color taking most of their surface area, see Fig. 1b and OSM Table 2), and the above-mentioned restrictions did not apply to them.

Each time a subject chose the positive stimulus in nine out of ten trials within a session, this session was considered successful and a new set of items was presented in the upcoming session (i.e., the subjects “moved on to the next set”; see Fig. 2). If out of five sets in a row four were successfully completed in the first session and one set was completed within the first two sessions, the subject was considered to have reached the learning criterion and moved on to *Phase 2* (see below, and Fig. 2).

#### Phase 2: Reversal learning

The procedure was similar to *Phase 1*, but only a single set of items was presented to the subjects (see Fig. 2). After an initial successful “acquisition” session (nine out of ten correct trials), the reward contingencies associated with each stimulus would be switched (i.e., the original positive stimulus becoming negative, and vice versa). Stimulus reward contingencies would be reversed after each successful session (nine out of ten correct trials; except for three occurrences in which a subject moved on to the next reversal with less correct trials due to human error – Aragorn’s tenth reversal and Amarok’s eighth reversal, with eight out of ten correct trials; and Nuru’s third reversal with seven out of ten). These reversals would take place until the learning criterion was achieved (equivalent to the one used for *Phase 1*: out of five consecutive reversals, four of them needed to be completed within the first session, and one within either the first or the second session).

### Data selection

The first 15 sets (*Phase 1*) were used for data analysis. We arrived at this number because one of the animals (Kaspar, one of the wolves) reached the learning criterion (and thus moved on to *Phase 2*) on the 15th set, with other animals reaching criterion a few sets afterwards (see OSM Table 1). Including data for more than 15 sets would thus incur the risk of biasing the results, as that would mean excluding the performance of the most successful animals (which would have moved to *Phase 2*; see OSM Table1 for more information on the performance of each subject). To make the size of the dataset similar to that of *Phase 1*, we used only the first 15 reversals for *Phase 2*. We considered this our initial dataset, and all of the additional data selection criteria described below were performed upon this dataset.

We removed all sessions in which experimental errors or anomalies took place from the analyses (4.8% of the dataset). Examples of these situations include sessions carried out after the 9/10 criterion was met (due to human error), sets that were not completed (because the items got broken), and sessions with more than two null trials (because the animals were not motivated to participate in the test). Whenever a session or a set was removed according to this criterion, the number of the session or set was transferred to the next valid one (e.g., if an animal’s fourth set was removed from the dataset, the following set would be considered set 4).

Furthermore, for a subset of the subjects in a reduced number of sessions (0.9% of the dataset), the procedure was considerably different (i.e., the subject stayed in a room adjacent to the testing chamber while E2 placed the items and would later be released back into the main testing chamber through a sliding door). These sessions were excluded from analyses as well.

Sessions that took place after February of 2016 were also excluded from the analyses (3.4% of the dataset) because testing became significantly less frequent after this date.

All sessions that were removed from analyses are still included in the dataset presented (Dataset 1; see also OSM Table  1 for more information on the performance of each subject), alongside the criterion that warranted their exclusion.

### Data analysis

We used R version 4.0.4 (R Core Team, 2021; https://www.r-project.org) to carry out all statistical analyses. For each experiment, two binomial generalized linear mixed models (GLMMs) with logit link function were conducted to analyze the data (lme4 package; Bates et al., 2015).

The response variable for the first group of models (Table 1, models 1 and 3) was the outcome of the session (i.e., whether the criterion of nine out of ten correct responses was met). The main predictors were the interaction between species and session (i.e., whether the species improved at a different speed within the sessions of the same set) and the interaction between species and set – in *Phase 1* – or reversal number – in *Phase 2* (i.e., whether the species improved at a different speed along the sets and within the same session in those sets). To account for the fact that sessions and sets had a nested structure (with each session being part of a single set/reversal), we created an ID for each session within a given set/reversal and included this variable (nested within set/reversal number) as a random intercept of the model. Finally, we also included the subjects as a random intercept.

Since these models did not distinguish between performances below the nine out of ten criterion (e.g., a session with eight out of ten correct trials would be considered no different from one with two out of ten correct trials), and in order to account for the fact that different subjects would perform a different number of trials within each set or reversal (as they needed more or less sessions to reach criterion within each set/reversal), we decided to fit a second group of models (Table 1, models 2 and 4). In these models, the response variable was the outcome of each trial (success or failure) within the first session of a given set/reversal, and the predictors were the interaction between species and trial (i.e., learning speed within each set for each species) and the interaction between species and set number (learning speed within each trial for each species). Similar to the previous set of models, we created an ID for each trial within each set/reversal and included it as a random intercept nested within the number of the set/reversal. The subjects were also included as a random intercept.

All numerical variables were z-transformed for model fitting. Random slopes were added whenever the variable had at least three different values per level of the respective random intercept (for continuous variables) or at least two levels with at least two observations per level of the respective random intercept (for discrete variables).

Each of these models was compared with a *null* version that had the same structure but excluded species as a variable. To account for the possibility that there were no differences in performance as a factor of improvement along trials, sessions, or sets/reversals, but still overall differences in performance between the species (as well as for the possibility that there are no differences between the species, but still variation along trials, sessions, or sets/reversals), a *reduced* version of the models (with the same predictors as the *full* models, but without the interaction) was also fitted and subsequently compared with the null as well (Table 1).

All models were tested for collinearity with the *vif* function from the *car* package (Fox & Weisberg, 2018) and further tested for model stability through a set of custom-made functions, designed by Roger Mundry and later edited by Remco Fokertsma, and the *DHARMa* library (Hartig, 2020). All models met stability and collinearity assumptions, with variance inflation factors (vif) under three in all cases (largest vif = 2.931; Field, 2009; Quinn & Keough, 2002).

### Ethics statement

No special permission for use of animals (wolves and dogs) in socio-cognitive studies is required by Austrian law (Tierversuchsgesetz 2012–TVG 2012). The relevant committee that allows running research without special permissions regarding animals is: Tierversuchskommission am Bundesministerium für Wissenschaft und Forschung (Austria). We followed ASAB (Guidelines for the Ethical Treatment of Nonhuman Animals in Behavioural Research and Teaching, 2023) guidelines for the ethical treatment of animals in behavioral research and teaching.

## Results

### Phase 1: Serial learning

Out of the original 22 dogs and 17 wolves; 15 dogs and 16 wolves participated in the study to at least set 15 (with the rest being taken out of the experiment due to unrelated factors; see Fig. 2 and OSM Table 1 for more details). Both species needed a similar number of sessions to reach set 15, with 43.3 ± 2.0 sessions on average for dogs, and 39.5 ± 2.7 sessions on average for wolves (t_(11.8)_ = 1.132, *p* = 0.280).

**Fig. 3:**
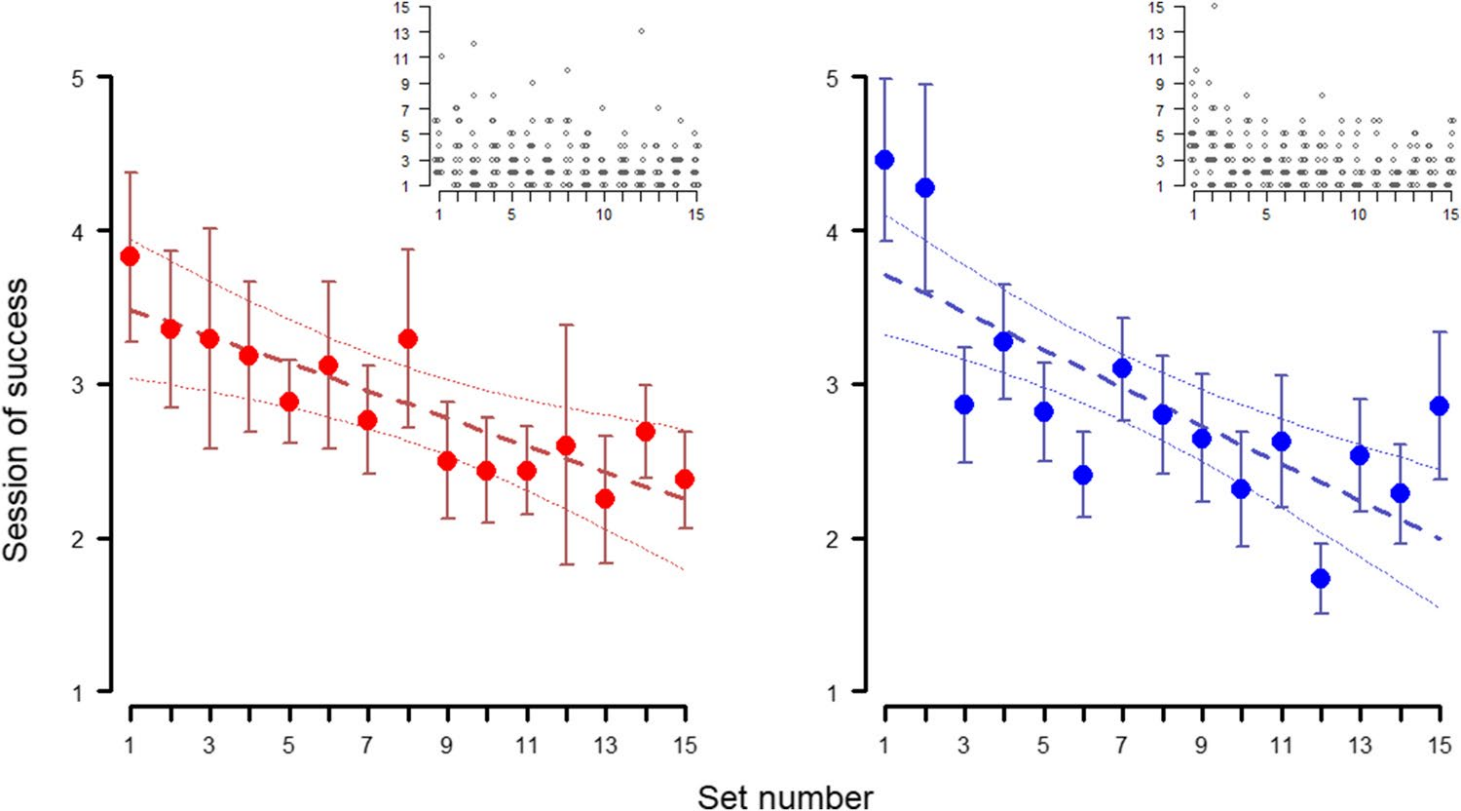
Across-session discrimination behavior - *Phase 1*. Average session of success at each set (at least nine out of ten correct trials) in *Phase 1* for wolves (red, left) and dogs (blue, right). Whiskers represent the standard error. Regression lines were calculated through a linear model with session of success as the dependent variable and set number as the independent variable, with confidence intervals set at 95%. Insets show individual data points

With each new set of objects presented, the subjects became faster at associating the food reward with choosing the positive stimulus (needing less sessions per set to reach the criterion; Fig. 3). However, we did not find differences between the species (full-null comparison Table 1: 1.1 and 1.3 : χ^2^ = 0.456, *p* = 0.928; reduced-null comparison Table 1: 1.2 and 1.3: χ^2^ = 0.221, *p* = 0.638). As for the null model, (Table 1: 1.3), both the effects of the session number (estimate ± SE: 0.547 ± 0.105; z value = 5.215, *p* < 0.001) and the set number (estimate ± SE: 0.303 ± 0.059; z value = 5.097, *p* < 0.001) were significant, showing that, indeed, subjects needed less sessions to reach criterion in later sets, and that each session within the same set increased the likelihood of success.

**Fig. 4:**
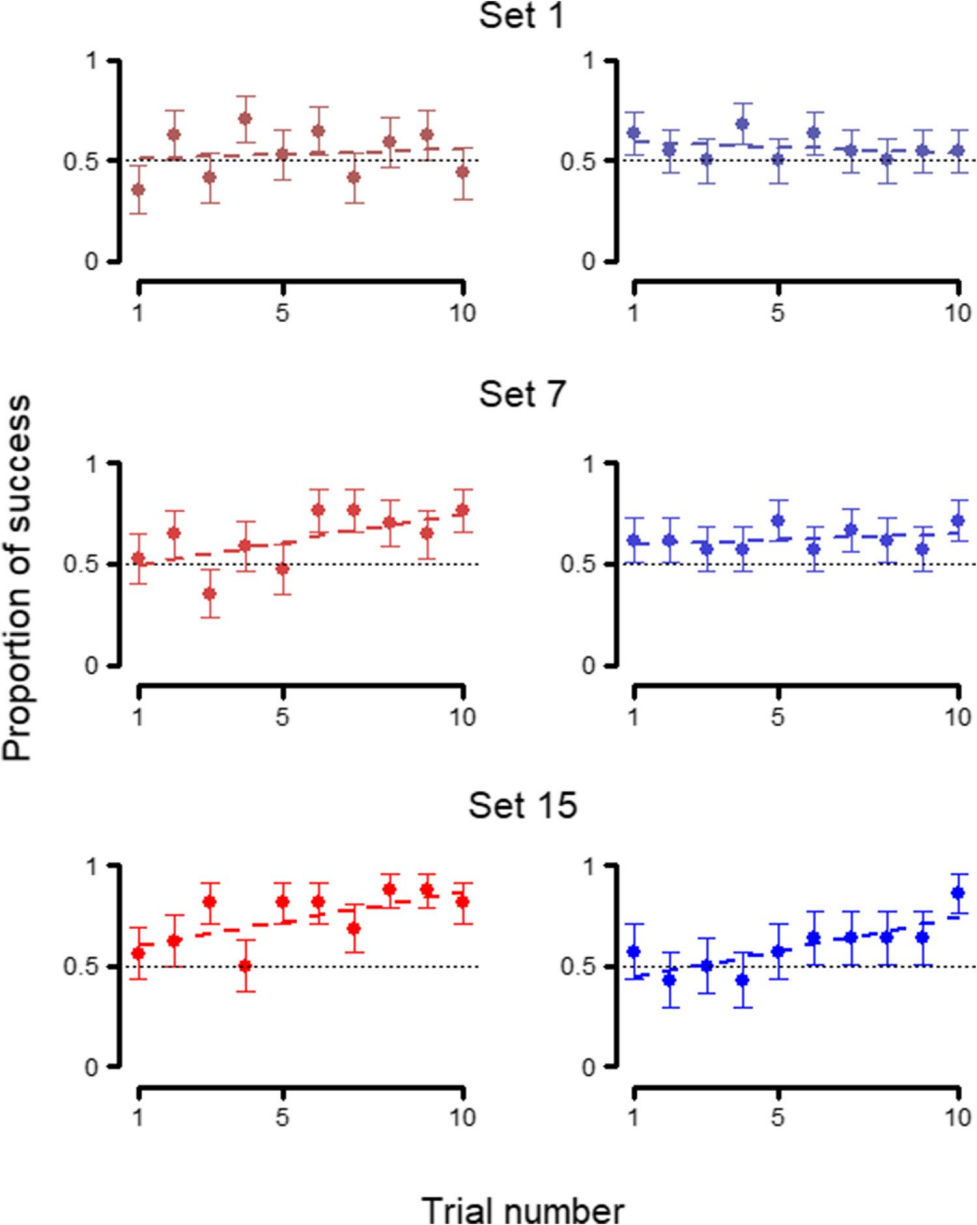
Trial-by-trial behavior at first session – *Phase 1*. Average performance in each trial of *Phase 1* at the first session of sets 1, 7, and 15, for wolves (left, red) and dogs (right, blue). Whiskers represent the standard error. Regression lines were calculated through a linear model with session of success as the dependent variable and trial number as the independent variable

When analyzing the outcome of each trial on the first session of each set (Table 1: 2.1), once again, we did not find an effect of the species (full-null comparison Table 1: 2.1 and 2.3: χ^2^ = 0.547, *p* = 0.908; reduced-null comparison Table 1: 2.2 and 2.3: χ^2^ = 0.517, *p* = 0.772), as can be seen in Fig. 4, for sets 1, 7, and 15. Similar to the previous model, both the trial number (estimate ± SE: 0.206 ± 0.022; z value = 9.454, *p* < 0.001) and the set number (estimate ± SE: 0.085 ± 0.031; z value = 2.778, *p* = 0.005) were significant, meaning that, when looking at the first session per set, both species improved along trials and sets.

### Phase 2: Reversal learning

All of the subjects that reached criterion in *Phase 1* (see Fig. 2 and OSM Table 1 for details) and continued on to *Phase 2* (six out of 22 dogs, eight out of 17 wolves, the rest either failing to reach this criterion within the timespan of the study or being taken out of the experiment due to unrelated circumstances; see OSM Table 1 for more details) had experienced a similar number of sets in *Phase 1*, with dogs going through an average of 32.8 ± 3.6 sets and wolves through an average of 34.0 ± 6.7 sets (t_(10.35)_ = -0.153, *p* = 0.881). They also had carried out a similar total number of sessions in *Phase 1* with 80.2 ± 7.4 sessions for dogs and 78.3 ± 9.5 sessions for wolves (t_(9.59)_ = 0.107, *p* = 0.917). Out of these subjects, three dogs and seven wolves completed 15 reversals, with the average number of sessions to do so being 35.7 ± 5.8 for the dogs and 47.3 ± 4.3 for the wolves (t_(4.30)_ = -1.614, *p* = 0.177).

**Fig. 5:**
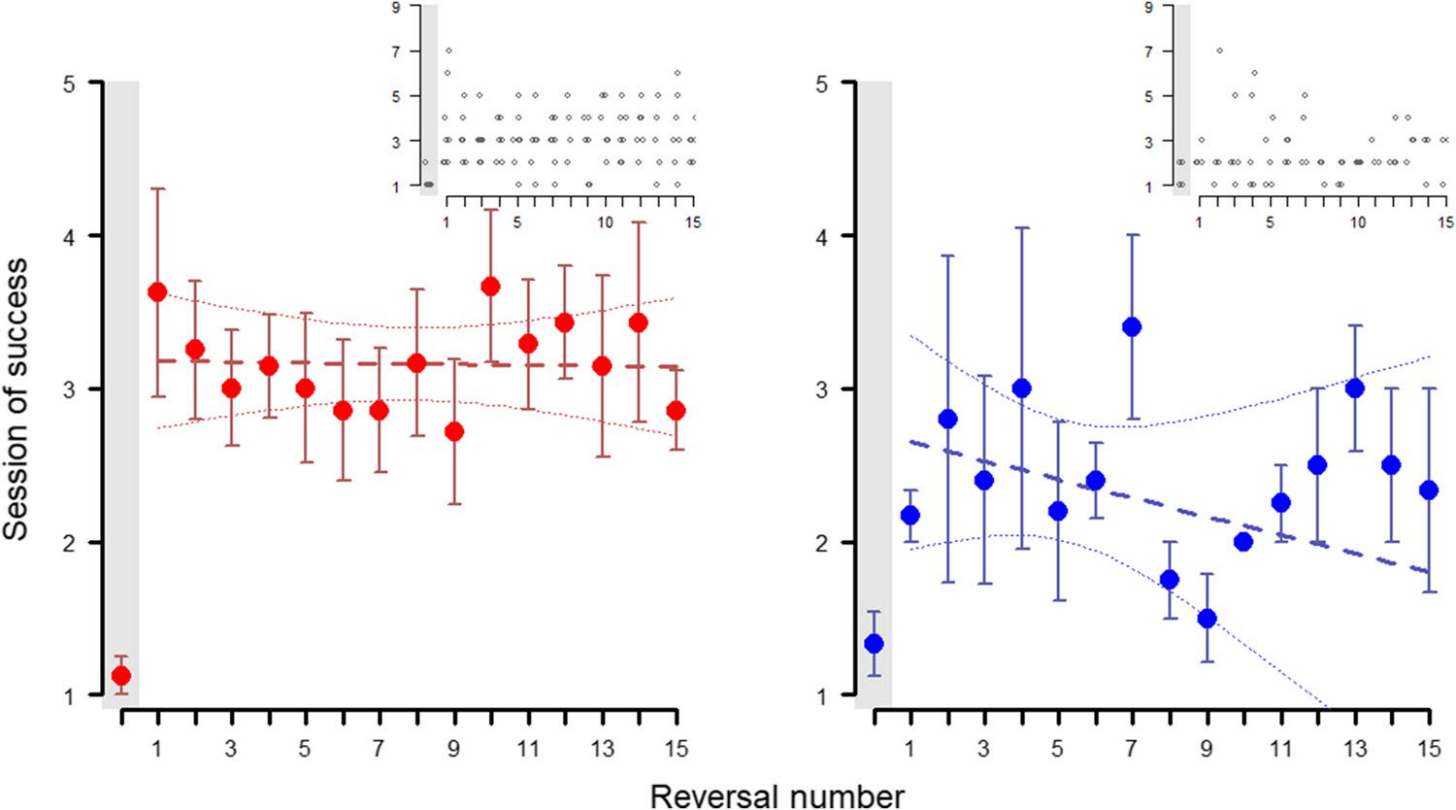
Across-sessions serial discrimination behavior – *Phase 2*. Average session of success at each reversal (at least nine out of ten correct trials) in *Phase 2* for the wolves (left, red) and the dogs (right, blue). “Reversal 0” (highlighted in gray) refers to the acquisition of the association, before the reward contingencies were reversed for the first time (functionally equivalent to one of the sets in *Phase 1*). Whiskers represent the standard error. Regression lines were calculated through a linear model with session of success as the dependent variable and reversal number as the independent variable, with confidence intervals set at 95%. Insets show the individual data points

Similar to *Phase 1*, we did not find any differences between the species when analyzing the session at which they reached criterion each time the reward contingencies were reversed (full-null comparison Table 1: 3.1 and 3.3: χ^2^ = 2.503, *p* = 0.475; reduced-null comparison Table 1: 3.1 and 3.3: χ^2^ = 0.799, *p* = 0.371). Furthermore, there was no apparent learning effect along successive reversals for either species (see Fig. 5). This was also shown in the models, as there was no significant effect of the reversal number in the null model (Table 1: 3.3: estimate ± SE: 0.041 ± 0.156; z value = 0.263, *p* = 0.793), although there was still an effect of the session number (estimate ± SE: 1.428 ± 0.170; z value = 8.359, *p* < 0.001). Taken together, these results indicate that the animals did not get significantly better with each reversal, or at least, that they did not improve enough with each reversal so as to reduce the number of sessions needed to reach the nine out of ten success criterion.

**Fig. 6:**
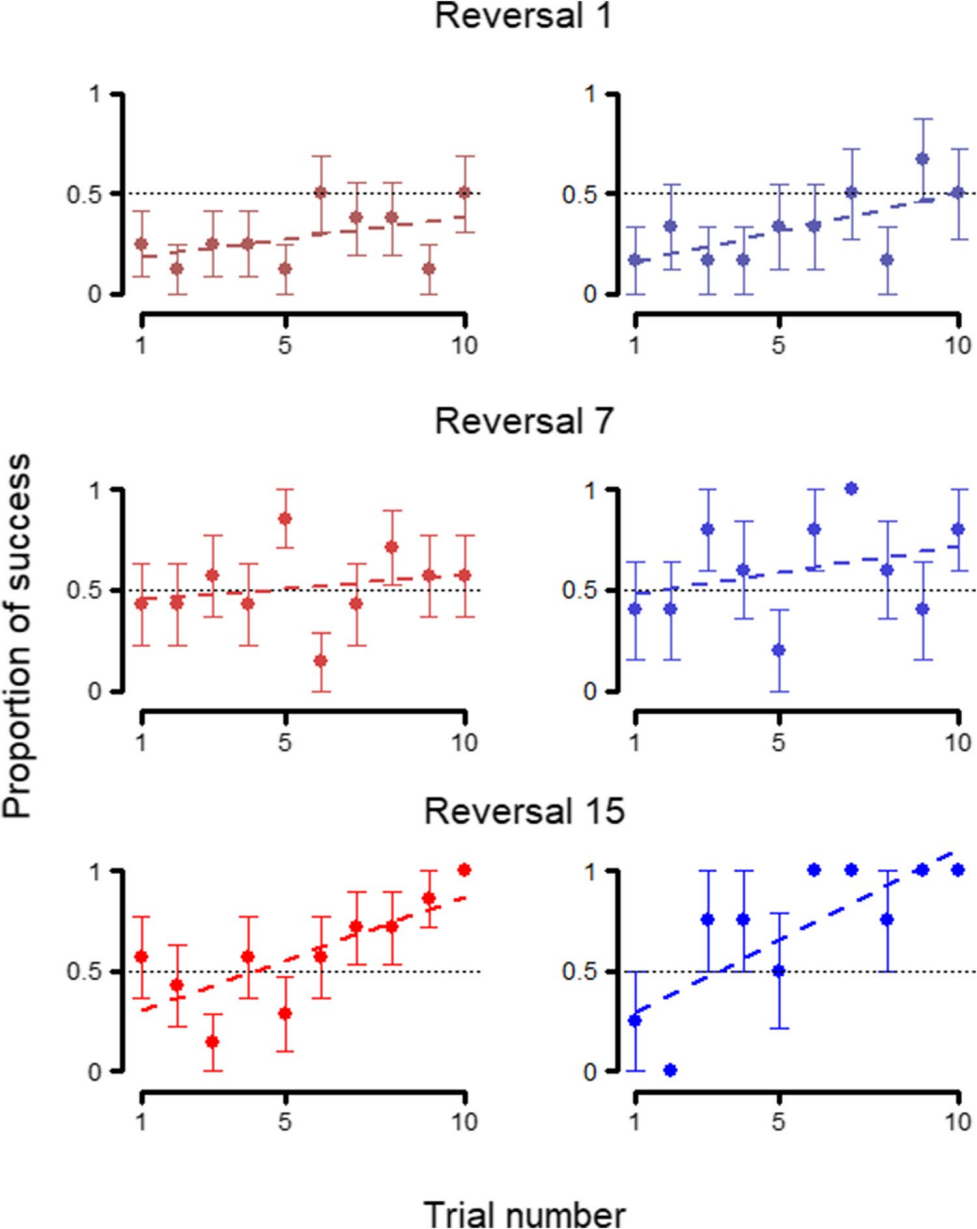
Trial-by-trial behavior at first session – *Phase 2*. Average performance in each trial of *Phase 2* at the first session of reversals 1, 7, and 15, for wolves (left, red) and dogs (right, blue) Whiskers represent the standard error. Regression lines were calculated through a linear model with session of success as the dependent variable and trial number as the independent variable

However, there was a significant effect of the species on the outcome of the trials on the first session of each reversal (full-null comparison Table 1: 4.1 and 4.3: χ^2^ = 12.977, *p* = 0.005), with the improvement with each passing trial being significantly worse in wolves (estimate ± SE: -0.158 ± 0.070; z value = -2.247, *p* = 0.025; Fig. 6), although the interaction between species and set number was not significant (estimate ± SE: 0.099 ± 0.080; z value = 1.235, *p* = 0.217).

We still found an effect of species when comparing this reduced model with the null model (reduced-null comparison Table 1**:** 4.2 and 4.3: χ^2^ = 6.336, *p* = 0.012; estimate ± SE: -0.279 ± 0.101; z value = -2.769, *p* = 0.006), and in this reduced model, also the trial number (estimate ± SE: 0.400 ± 0.034; z value = 11.690, *p* < 0.001) and the reversal number (estimate ± SE: 0.191 ± 0.049; z value = 3.877, *p* < 0.001) were significant; suggesting that the subjects’ performance on the first session did improve each time the reward contingencies were reversed.

## Discussion

In this study we examined wolves' and dogs' serial learning skills in both a serial discrimination and a reversal learning task. We did not find any differences between the species in *Phase 1* – serial discrimination learning, but the performance of each species did improve with each additional set of stimuli presented, showing that they both had “learnt to learn.” However, dogs did perform better than wolves in *Phase 2* – reversal learning, where the task was to extinguish learned associations and instead acquire an association with the previously unrewarded stimulus. Notwithstanding, dogs only outperformed wolves when analyzing the first session of each reversal, but no differences were found between the species along reversals. Similarly, dogs did not reach session criterion within each reversal faster than wolves, and neither species showed improvement at the session level (i.e., they did not need fewer sessions to achieve criterion with each reversal). Still, performance in the first session of each set starts as indistinguishable from chance in trial 1 and then rises to around 75% by set 15, for both species.

When compared to Harlow’s original study with the Rhesus monkeys, we did, then, not find the nigh-perfect performance at trial two that he reported (Harlow, 1949). However, this is, in all likelihood, a result of the reduced number of sets used in our study, when compared with Harlow’s 344 sets. Our number of sets was more comparable with Zeigler's (1961) experiment on pigeons and Arnold's (1957) experiments on rats, with performances above chance, but nevertheless not perfect by the second trial of each set. Nonetheless, even though comparisons between Harlow’s and our results are complicated (not only because of the differences in methodology, but also the manner in which the data were reported in the original study), it could be argued that, all things being equal, the performance by set 15 is not qualitatively dissimilar between our study and Harlow’s. In Harlow’s study, the average performance in sets 9–16 describes a mostly linear curve starting at 50% of correct choices and rising to somewhat above 80% by trial 6, while in ours it reaches around 75% by the 15th set. Of course, it is not possible to know whether increasing the number of trials would have led to a nigh-perfect performance by the end of the experiment in canids (particularly considering that higher testing volumes would not be possible under the current experimental conditions).

When considering only *Phase 2* of the experiment, it is hard to determine to which degree our results may have been influenced by our reduced sample size (eight wolves and six dogs on the first reversal, seven wolves and three dogs by the 15th one), and so conclusions derived from this part of the experiment should be examined with caution. Even so, the fact that dogs outperformed wolves in this phase remains an interesting outcome, as it seems to point towards dogs being more flexible than wolves (differences between the species were found only in the reversal learning phase – with reversal learning being a measurement of behavioral flexibility (Bond et al., 2007; Izquierdo et al., 2017). This aligns with the human-driven flexibility hypothesis, which predicts dogs are more adept at discarding associations and making new ones (Sih et al., 2011; but see Vincze & Kovács, 2022). Arguably, human-shaped environments, while usually plentiful in resources for free-ranging dogs, also present a higher degree of instability when compared to the more natural environments that wolves inhabit, as human activity can easily change the availability of resources (chiefly in this case, waste) in unpredictable patterns (as opposed to the availability of prey in the case of wolves, which answers to seasonal cycles; Metz et al., 2012; Sarkar et al., 2023). Furthermore, unlike wolves who live as cooperative hunters (in packs in which the higher-ranked individuals are highly tolerant of the subordinates eating in their presence), dogs live in fission-fusion societies with steep hierarchies (Range & Marshall-Pescini, 2022a), and thus, higher-ranked individuals potentially monopolize resources, making for a more complex foraging landscape both in time and space (something that is currently being investigated by our research group – Berghänel et al., 2022; Range et al., 2015).

We cannot exclude the possibility that dogs are, as predicted by the tractability hypothesis –which postulates that, as dogs were selected to perform many different tasks for humans (Frank, 2011) – better at making arbitrary associations, even though our findings offer limited support for this (as the hypothesis would predict that dogs outperform wolves in both phases of the experiment). However, it could be the case that dogs are indeed better at making arbitrary associations for which wolves can compensate with their higher persistence. Wolves have been shown to be more persistent than dogs, in that they engage in problem-solving tasks for longer and they are not deterred even when no solution is available (Rao et al., 2018). Nevertheless, in the case of the current study, this higher level of engagement would not provide a further advantage for wolves, as the number of trials was limited to 10, and the sessions in which the individuals were not motivated were not taken into consideration for the analyses (see the *Data selection* subsection in the *Methods*). Moreover, in the case of *Phase 2*, persistence could also lead wolves to continuing to choose the same incorrect object (Chow et al., 2016) – and they did indeed make more mistakes than dogs when only the first session was considered.

Nevertheless, it remains unclear why the observed increase in dogs’ performance in the first sessions of each reversal did not translate into differences in learning speed between the species overall. One explanation for the diverging results of two models used to analyze the data in *Phase 2* would be the steep success criterion. As only one mistake was allowed in order to proceed to the next reversal, better performances within each session would not necessarily mean achieving the criterion faster. Non-successful sessions did range from 0 to 8 out of 10 correct trials (as opposed to the 9 or 10 out of 10 correct trials needed for a successful session), which could mean that differences in learning speed could have been obscured by the success criterion. This may have been compounded by the fact that the task was more difficult than the one in *Phase 1*, which could explain why such an effect was not an issue there. Indeed, neither species showed a decrease in sessions needed to reach criterion within a reversal even though they did get better at solving the task within the first session of each successive reversal, which may point to a possible floor effect derived from the criterion we used. However, as previously stated, it is important to take into consideration the possibility of the observed results being an artifact of the reduced number of subjects in *Phase 2*.

Regardless of the reason behind the inconsistency between the first session and overall performance within *Phase 2*, it is also unclear what cognitive processes could underlie the observed differences in reversal learning between the species. In our task, in order to acquire a new and opposite association, the previous one would have to be extinguished, and in order to do so, the previous response towards the positive stimulus would need to be inhibited (Shettleworth, 2010). In light of our results, it would be plausible that either the dogs were better than the wolves at inhibiting the learned association, or that the dogs made weaker associations (i.e., they did not remember the reward contingencies as well as the wolves did), and thus had an easier time “un-learning” them. However, we cannot tease these possibilities apart within the confines of the current study, as we did not include any controlled measures of memory in our paradigm. Dogs have been shown to retain learned associations for a period of at least 6 months (Wallis et al., 2016), making it unlikely that the observed results may come as a consequence of poor memory. Additionally, no differences in inhibition have been found between dogs and wolves (or, when they have, they were not extrapolatable across tasks (Brucks et al., 2019; Wallis et al., 2016), making it hard to draw any conclusions about differences in the capability to inhibit learned associations. Thus, future studies need to measure both instrumental learning and memory capabilities of dogs and wolves in a controlled manner (e.g., as part of a battery of tests).

Comparative studies on learning in wolves and dogs remain scarce, with our results still failing to provide a clearer picture when put into context with the current literature (Miklosi, 2007; Range & Marshall-Pescini, 2022b). This further highlights the relevance of carrying out more experiments on this topic, as it is now apparent that much is still unknown about such an important process, which could prove fundamental to other studies performed on these species. It is our view that, without a solid grasp of how instrumental learning works in these species, the efforts towards studying other, more complex, behaviors that may rely on it may prove to be misguided.

Still, it is also important to mention that despite our reduced sample size (seven wolves and three dogs by the end of *Phase 2*) – which increases the possibility that the differences observed may have come as a result of a statistical artifact – unfortunately it was not possible to increase the number of subjects who participated in this experiment beyond what was used. Similarly raised wolves and dogs are an irreplaceable resource; raising them up to the point at which they are fit to be tested in most behavioral experiments takes roughly a year. Further than that, getting to the second phase of this study required tens of sessions for most animals, further increasing the difficulty of testing a larger sample size. Ultimately, for practical reasons, increasing the number of animals is not possible within the available infrastructure.

As a way to tackle the above-mentioned issues and further explore the topic at hand, one possible avenue to continue studying instrumental learning in wolves and dogs would be to employ methods such as touchscreens or other automatized apparatuses. Possibly because the subjects do not need to be repositioned after each trial and the fact that the reward is dispensed automatically, touchscreen-based paradigms have been fairly successful in carrying out large volumes of trials in dogs and wolves (e.g., Aust et al., 2008; Dale et al., 2019; Laude et al., 2016; Range et al., 2008; Rivas-Blanco et al., 2020), which also have the added advantage of increasing the number of animals who make it to the last part of the experiment. As all computer-based tasks need an instrumental learning training phase in order to teach the animals how to interact with the apparatus (usually by presenting them with a positive stimulus that is rewarded and a negative stimulus that is not; see Rivas-Blanco et al., 2020), it should be fairly straightforward to extend this training regime to further sets of images.

In conclusion, we found that both wolves and dogs improve with repeated exposure to the paradigm (although never at near-perfect levels by trial 2), but roughly at the same speed. Dogs may outperform wolves when it comes to extinguishing previously made associations and learning new ones in their stead, but more research needs to be done on the matter. Further studies are needed that consider the use of automatized testing methods, as they allow for a higher testing volume while reducing the subjects’ fatigue, which in turn would reduce the possibility of needing to take animals out of the experiment (due to unrelated circumstances derived from long study periods). Moreover, these methods would contribute to reducing experimental running times and human-driven errors, improving standardization of procedures and ultimately increasing reproducibility.

## Supplementary Information

Below is the link to the electronic supplementary material.

Supplementary file1 (PDF 72 KB)

Supplementary file2 (PDF 57 KB)

## Data Availability

All data generated or analyzed during this study are included in this published article as supplementary materials.
