# Supplementary material for: Going back to “basics”: Harlow’s learning set task with wolves and dogs
Source: Learn Behav. 2024 May 23;52(4):315–29. doi: 10.3758/s13420-024-00631-6 (PMC11628440; doi:10.3758/s13420-024-00631-6)
Supplement: Supplementary file 1 — Supplementary file1 (PDF 72 KB) [file 13420_2024_631_MOESM1_ESM.pdf]

Supplementary Table 1: List of all the subjects that participated in this study, with their species and sex, whether they participated only in Experiment 1 or both, their age at the beginning of Phase 1 (and Phase 2, whenever it applied) and the amount of sets and reversals they were exposed to in each experimental phase. Subjects marked in gray fulfilled the criterion of either the Phase 1 (whenever the grayed out area reached the third-to-last column) or both experimental phases (whenever the whole row is grayed out).

Name	Species	Sex	Phases	Age at Phase 1 (months)	Last Phase 1 set	Age at Phase 2 (months)	Last Phase 2 reversal
**Alika**	dog	f	Phase 1	10	8		
**Asali**	dog	m	Phase 1 & Phase 2	13	45		
**Banzai**	dog	m	Phase 1	9	9		
**Bashira**	dog	f	Phase 1	13	16		
**Binti**	dog	f	Phase 1 & Phase 2	13	28	35	37
**Bora**	dog	f	Phase 1	12	54		
**Enzi**	dog	m	Phase 1	9	9		
**Gombo**	dog	m	Phase 1	10	15		
**Hakima**	dog	m	Phase 1 & Phase 2	13	28	33	2
**Hiari**	dog	m	Phase 1	10	17		
**Imara**	dog	f	Phase 1	10	10		
**Kilio**	dog	m	Phase 1 & Phase 2	9	31	22	29
**Layla**	dog	f	Phase 1	12	43		
**Maisha**	dog	m	Phase 1 & Phase 2	9	25	21	72
**Meru**	dog	m	Phase 1 & Phase 2	22	49	52	7
**Nia**	dog	f	Phase 1	10	51		
**Nuru**	dog	m	Phase 1 & Phase 2	10	36	40	14
**Panya**	dog	f	Phase 1	22	12		
**Pepeo**	dog	m	Phase 1	23	9		
**Rafiki**	dog	m	Phase 1	61	55		
**Sahibu**	dog	m	Phase 1	10	6		
**Zuri**	dog	f	Phase 1	10	60		
**Amarok**	wolf	m	Phase 1 & Phase 2	10	20	29	24
**Apache**	wolf	m	Phase 1 & Phase 2	14	40	34	3
**Aragorn**	wolf	m	Phase 1 & Phase 2	9	15	19	88
**Cherokee**	wolf	m	Phase 1	10	47		
**Chitto**	wolf	m	Phase 1	10	24		
**Geronimo**	wolf	m	Phase 1 & Phase 2	10	63	43	32
**Kaspar**	wolf	m	Phase 1 & Phase 2	9	16	13	144
**Kay**	wolf	f	Phase 1	10	9		

**Kenai**	wolf	m	Phase 1 & Phase 2	11	41	38	40
**Nanuk**	wolf	m	Phase 1	17	68		
**Shima**	wolf	f	Phase 1 & Phase 2	29	57	28	80
**Tala**	wolf	f	Phase 1	29	45		
**Tatonga**	wolf	f	Phase 1	45	50		
**Una**	wolf	f	Phase 1	9	20		
**Wamblee***	wolf	m	Phase 1	11	29		
**Wapi**	wolf	m	Phase 1	11	38		
**Yukon**	wolf	f	Phase 1 & Phase 2	15	20	23	46

*: Date for first session not recorded, date for second session used to calculate age.
