# Supplementary material for: Going back to “basics”: Harlow’s learning set task with wolves and dogs
Source: Learn Behav. 2024 May 23;52(4):315–29. doi: 10.3758/s13420-024-00631-6 (PMC11628440; doi:10.3758/s13420-024-00631-6)
Supplement: Supplementary file 2 — Supplementary file2 (PDF 57 KB) [file 13420_2024_631_MOESM2_ESM.pdf]

Supplementary Table 2: IDs of the objects used in this experiment, together with their colors.

**Item**	**Color**
pink_barbie_box	pink
pink_barbie_box_lid	pink
black_ashtray	black
black_cofee_pot	black
black_cup	black
black_devil	black
black_filter	black
black_wheel	black
blue_castle	blue
blue_filter	blue
blue_havaball	blue
blue_pretzel	blue
blue_seahorse	blue
blue_strawberry	blue
blue_train	blue
brown_cappuccino_box	brown
brown_leaf	brown
brown_pot	brown
brown_cardboard_box	brown
white_pot_lid	white
cookie_box_lid	mixed
yellow_pedros_lid	yellow
green_croissant	green
green_face	green
green_plane	green
green_shovel	green
green_soap_frog	green
green_whale	green
grey_egg_holder	grey
grey_rubber_dogbasket_foot	grey
ice_cream_pot	mixed
silver_cup	silver
orange_castle	orange
orange_face	orange
orange_ovomaltine_box	orange
purple_big_half_ball	purple
pink_bowl	pink
purple_half_ball	purple
purple_multivitamin_tube	purple

red_bread	red
red_frisbee	red
red_hand	red
red_toy_horse	red
red_pear	red
red_shark	red
red_train	red
santa	mixed
silver_shopping_cart_wheel	silver
silver_coffee_pad	silver
silver_dish	silver
silver_pot	silver
silver_tube	silver
stone	mixed
white_dish_with_hole	white
white_flower_dish	white
white_half_ball	white
white_star	white
yellow_bin_top	yellow
yellow_bowl	yellow
yellow_ice_cream	yellow
yellow_plane	yellow
yellow_ship	yellow
yellow_star	yellow
yellow_tree	yellow
blue_foot	blue
blue_pot	blue
blue_shell	blue
blue_tower	blue
brown_flower_dish	brown
erdal_lid	mixed
golden_lid	golden
green_apple	green
green_half_ball	green
grey_manner_minder_front	grey
orange_lid	orange
orange_tower	orange
pink_crab	pink
pink_half_ball	pink
pink_seahorse	pink
red_cup	red
white_ashtray	white

white_box	white
yellow_egg_holder	yellow
yellow_shovel	yellow
yellow_strawberry	yellow
green_aloe_vera_box	green
black_coffee_filter	black
blue_berry	blue
blue_crab	blue
blue_cup	blue
brown_pen_holder	brown
golden_cup	golden
green_pot	green
green_banana	green
light_blue_train	blue
silver_coffee_pot	silver
pink_ship	pink
red_tube	red
silver_tape_(seminar_room)	silver
white_orbit_box	white
white_wheel	white
yellow_lid	yellow
yellow_lobster	yellow
light_blue_tower	blue
silver_coffee_box	silver
brown_flower_pot	brown
